# Venus: An efficient *v*irus infection d*e*tectio*n* and f*u*sion *s*ite discovery method using single-cell and bulk RNA-seq data

**DOI:** 10.1371/journal.pcbi.1010636

**Published:** 2022-10-27

**Authors:** Che Yu Lee, Yuhang Chen, Ziheng Duan, Min Xu, Matthew J. Girgenti, Ke Xu, Mark Gerstein, Jing Zhang

**Affiliations:** 1 Department of Computer Science, University of California, Irvine, California, United States of America; 2 Computational Biology & Bioinformatics Program, Yale University, New Haven, Connecticut, United States of America; 3 Computational Biology Department, Carnegie Mellon University, Pittsburgh, Pennsylvania, United States of America; 4 Department of Psychiatry, School of Medicine, Yale University, New Haven, Connecticut, United States of America; 5 Clinical Neurosciences Division, National Center for PTSD, U.S. Department of Veterans Affairs, West Haven, Connecticut, United States of America; 6 Connecticut Veteran Healthcare System, West Haven, Connecticut, United States of America; 7 Molecular Biophysics & Biochemistry, Yale University, New Haven, Connecticut, United States of America; University of Toronto, CANADA

## Abstract

Early and accurate detection of viruses in clinical and environmental samples is essential for effective public healthcare, treatment, and therapeutics. While PCR detects potential pathogens with high sensitivity, it is difficult to scale and requires knowledge of the exact sequence of the pathogen. With the advent of next-gen single-cell sequencing, it is now possible to scrutinize viral transcriptomics at the finest possible resolution–cells. This newfound ability to investigate individual cells opens new avenues to understand viral pathophysiology with unprecedented resolution. To leverage this ability, we propose an efficient and accurate computational pipeline, named Venus, for virus detection and integration site discovery in both single-cell and bulk-tissue RNA-seq data. Specifically, Venus addresses two main questions: whether a tissue/cell type is infected by viruses or a virus of interest? And if infected, whether and where has the virus inserted itself into the human genome? Our analysis can be broken into two parts–validation and discovery. Firstly, for validation, we applied Venus on well-studied viral datasets, such as HBV- hepatocellular carcinoma and HIV-infection treated with antiretroviral therapy. Secondly, for discovery, we analyzed datasets such as HIV-infected neurological patients and deeply sequenced T-cells. We detected viral transcripts in the novel target of the brain and high-confidence integration sites in immune cells. In conclusion, here we describe Venus, a publicly available software which we believe will be a valuable virus investigation tool for the scientific community at large.

This is a *PLOS Computational Biology* Software paper.

## Introduction

Viruses pose a significant threat to humanity, ranging from the common cold to the recent global pandemic. For instance, they account for 12% of all human cancers and countless of human deaths [1]. Their complex interplay with viral host has made most cures elusive for scientists. Much like previous viral epidemics HIV/AIDS, MERS, and EBOLA, the world is currently struggling through a once-in-a-century pandemic SARS-CoV-2 that has claimed half a million American lives and five million globally, showing the political and economic repercussions of a viral epidemic [2]. Indeed, viral diseases are of major significance not only to science but also to society at large.

Several methods have been developed to dissect the virus-host interactome. Utilizing computational subtraction on high-throughput sequencing data, they detect viral reads and identify specific virus species to investigate the molecular mechanisms of certain viral-caused diseases, such as HBV’s hepatocellular carcinoma and HIV’s immune deficiency. For instance, SRSA, VirTect, PathSeq, and VirusSeq search for virus-specific transcripts in bulk RNA-seq reads [3–6]. While promising in detecting viruses, they are designed for reads pooled from thousands to millions of heterogeneous cells of complex tissues. Thus, even after successful mapping, one conundrum remains: which specific cell types are these viruses targeting.

**Fig 1:**
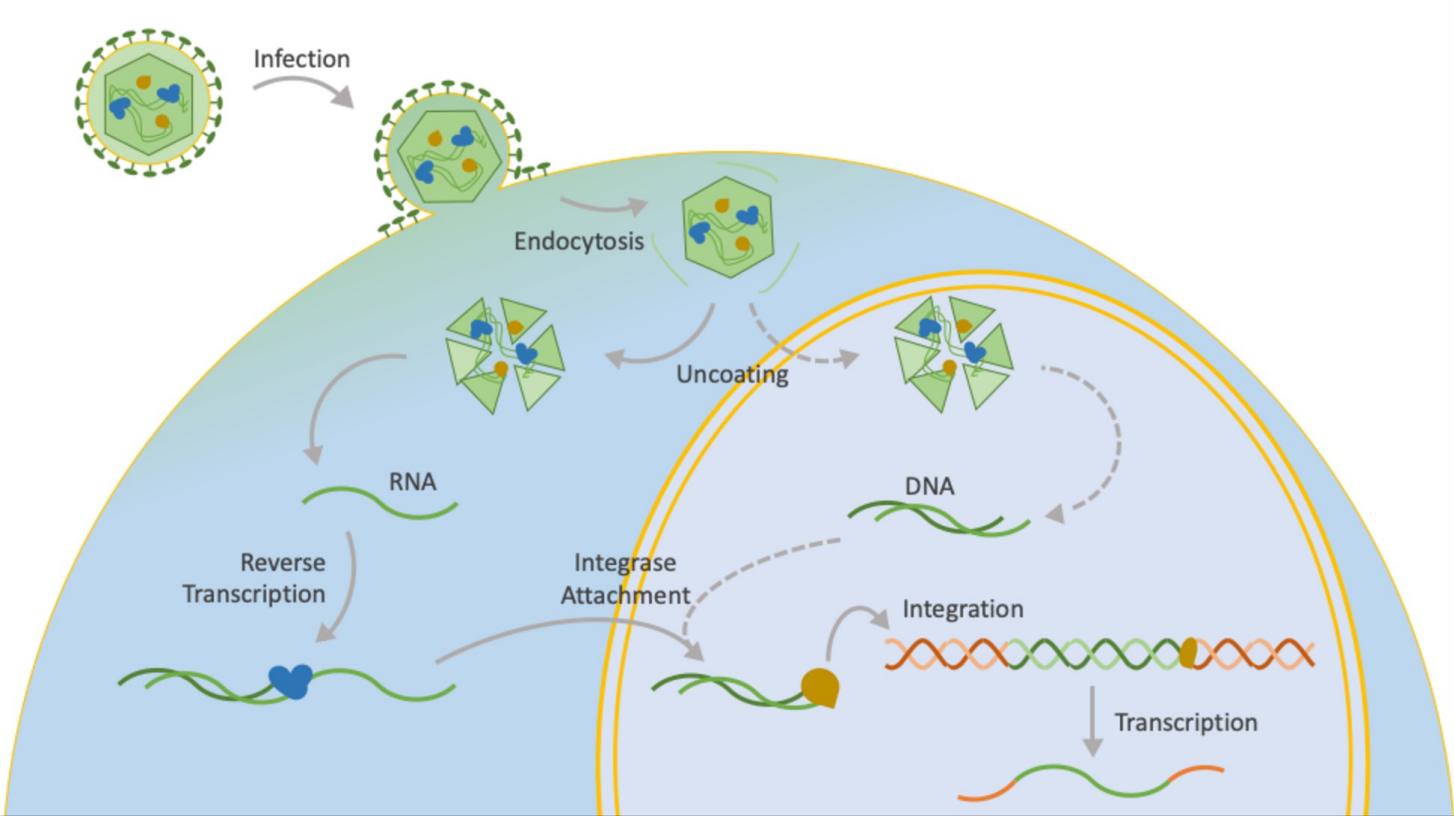
Biological schematic of virus integration. DNA viruses are indicated in dashed arrows, while RNA retroviruses and processes common to both are indicated in solid arrows. Inspiration for our hand-drawn figure is linked here (www.britannica.com/science/reverse-transcriptase) [13].

Recent advances in single-cell RNA sequencing technologies [7,8] have allowed us to simultaneously capture transcripts in millions of cells, providing the opportunity to dissect the transcriptome at a single cell resolution. Thus, it is now possible to characterize the virus-host interactome in individual cells. While several recent computational methods were developed to study viruses at a single-cell resolution [9–11], they failed to identify the many integration-able viruses and report virus integration sites (**Fig 1**). Such an answer is valuable, because integration sites contribute to cell death, tumorigenesis, viral persistence, and even variant evolution [12].

**Fig 2:**
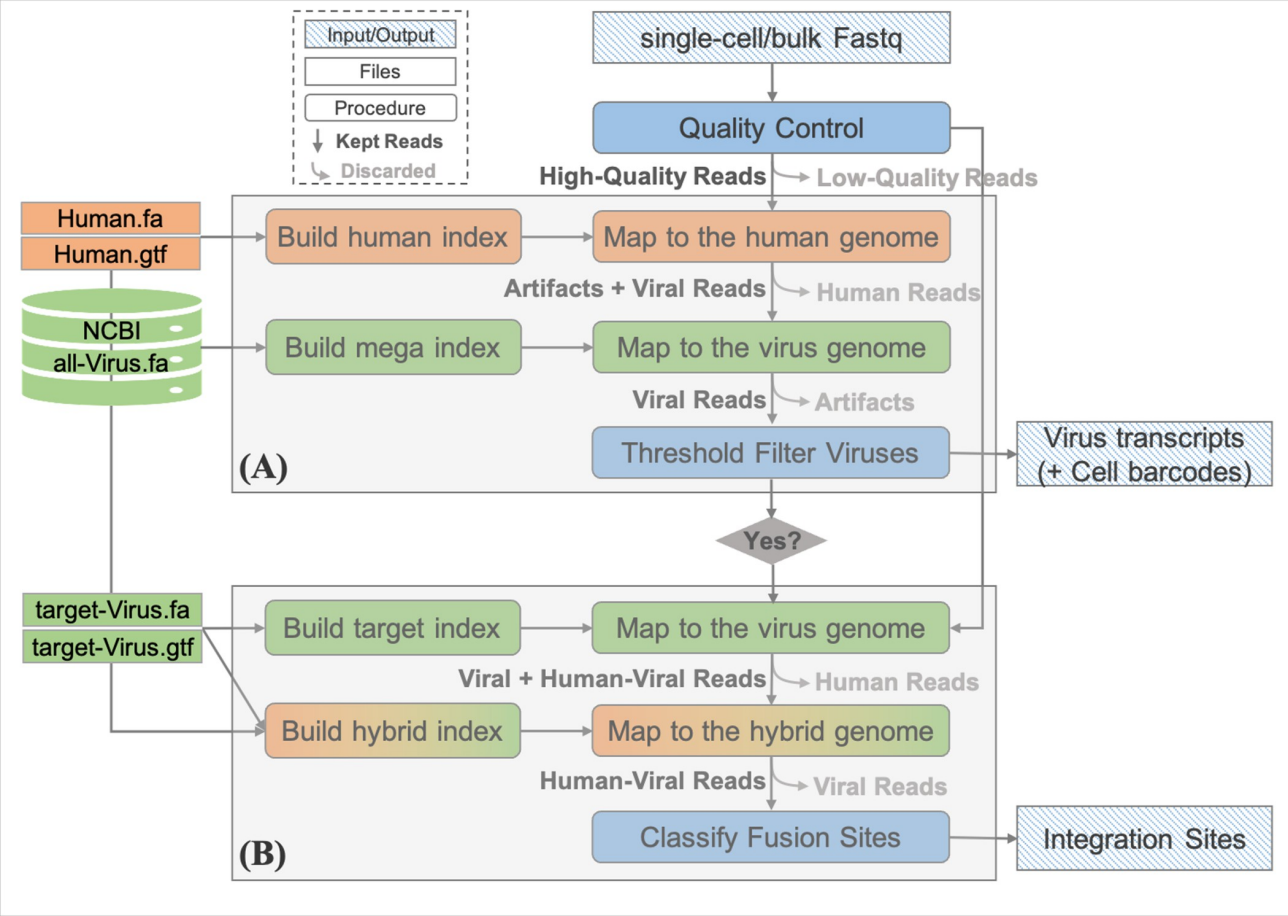
Venus’s workflow. **(A)** Virus detection module: a subtractive analysis that first aligns reads to the human genome and then maps the unmapped reads to the viral genome. **(B)** Integration site discovery module: a recycling process that first aligns reads to a target viral genome and then maps the mapped reads to a hybrid genome.

To address the aforementioned challenges, we developed Venus, an efficient Virus infection and fusion site detection method for both bulk-tissue and single-cell RNA-seq data (**Fig 2**). We demonstrated Venus’s two modules–detection and integration–on four public RNA-seq datasets, one of which was Hepatitis B Virus-infected (HBV) liver cancer while the other three were Human Immunodeficiency Virus-infected (HIV) monocytes, brain, and T-cells. Firstly, for the detection module, we validated Venus’s accuracy and single-cell capability by detecting 95% HBV infection in the liver cancer dataset and labeling HIV-infection at a single-cell resolution in the monocyte dataset. Venus even discovered a novel target of HIV by reporting infection in the human frontal cortex in the HIV-infected brain dataset. Secondly, for the integration module, Venus identified 52 fusion sites over 18 chromosomes in the HBV liver cancer dataset and around 6000 fusion sites in the HIV T-cell datasets. Utilizing a biology-based classification technique and visualization, Venus diminished the number of HIV T-cell fusion sites down to 17 high-confidence full length integration sites. All in all, Venus discovered infected cell types, novel viral targets, and meaningful integration sites across multiple virus-infected datasets.

## Design and implementation

### Overall Venus work flow

Venus is an efficient computational *software* pipeline for virus detection and integration site discovery for both single-cell and bulk transcriptomic data. Venus consisted of two main modules: virus detection and integration site discovery. The recommended guideline is to always run the virus detection module but only run the integration module if the virus species is able to integrate its genomic information into the host. Each module is described in detail below.

### Virus detection module in Venus

Venus utilized a sequential analysis to detect viruses (**Fig 2A**). It first aligned reads to the human genome and then aligned the leftover unmapped reads to a mega-viral genome. Finally, the virusThreshold parameter removed viral species with low number of supporting reads (**Table 1**). What is most important will be the threshold set for transcript filtering. We recommend starting with a threshold of zero first and then deciding on a new threshold with the results. For single-cell data, barcode and UMI were specified while a whitelist was inputted if available.

**Table 1:** Venus’s detection module parameters.

Option	Description
--reads read_1.fastq read_2.fastq	Reads
--virusThreshold 5 --virusChrRef virus_chr-ref.tsv	Virus threshold for filtering NCBI accession to species metadata file
--virusGenome virus.genomeDir --humanGenome human.genomeDir	Genome indices directories created to map our reads
--singleCellBarcode 1, 16 --singleUniqueMolIdent 17, 12 --singleWhitelist whitelist.txt	Specifications for single-cell data Numbers represent position, length, respectively
--out path/to/output/dir --readFilesCommand zcat --thread 32	General parameters

Human genome (version GRCh38.p13) and annotation file (version GRCh38.p13) were download from the GENCODE website. 7571 viral genomes were downloaded from NCBI and then concatenated to make the mega-virus index (annotation files were unavailable). Indices and reads were built and mapped using STAR version 2.7.9a [14].

### Integration site detection module in Venus

After detecting the virus of interest (target virus), we further developed efficient pipelines for integration site discovery. Specifically, Venus contained three steps for accurate integration site detection, as shown in **Fig 2B**. Parameters used are described and bolded in **Table 2**. What is most important in the integration module will be the integrSeq.fna file, which contains biological sequences Venus should specifically look for in its fusion sites to classify meaningful integration sites. For HIV and other retroviruses, this will be the LTR sequences. Firstly, Venus selected the reads mappable to the target virus genome as the starting point for maximum processing efficiency because viruses have smaller genomes than humans and mapping first to the virus genome without splicing increases detection sensitivity. Secondly, the virus-mappable reads were then mapped with splicing to a custom hybrid genome, made from concatenating human and target viral fasta/gtf files. Thirdly, chimeric fusion transcripts were sorted and classified based on the integrSeq parameter to provide biologically relevant integration sites.

**Table 2:** Venus’s integration site discovery module parameters.

Option	Description
--reads read_1.fastq read_2.fastq	Reads, should only be cDNA reads (no barcodes/UMI)
--guideFASTA integrSeq.fa --geneBed genes.bed --virusChr NC_001802.1	*integrSeq*.*fa* are sequences for fusion site classification. *genes*.*bed* converts genomic coordinates to genes NCBI virus accession id
--virusGenome virus.genomeDir --hybridGenome hybrid.genomeDir	Genome indices directories created to map our reads
--out path/to/output/dir --readFilesCommand zcat --thread 32	General parameters

### Classification of fusion transcripts into different confidence-level integration sites

Based on the user-defined parameter integrSeq, Venus classified its chimeric fusion transcripts by biological significance. The parameter integrSeq was put in place because only full viral integrations as opposed to partial ones were biologically important. Many integrated viruses contain conserved flanking sequences, such as the long-terminal repeats (LTR) in all RNA retroviruses [15,16], to help guide this classification.

To detect biologically significant sites, Venus mapped to the integrSeq sequence. Venus also ensured that each chimeric read had a clear junction breakpoint, with no gaps or overlaps between the two portions, a quality of true integration sites [17]. Fusion transcripts were then sorted into classes based on integration locations on the human genome. A final IGV-compatible visualization file was provided for manual validation.

When classifying fusion sites, the integrSeq parameter supplied the necessary viral promoter and terminator sequences. Fusion sites qualified for integration site:

- Class I) if they had human reading into the viral promoter sequence, had viral terminator reading into human sequences, or had known splice sites from both species;
- Class II) if they had either the above-mentioned viral promoter or terminator sequences but read from or into noncoding human regions, respectively;
- Class III) if they mapped to middle of viral genes.

### Bulk and single-cell RNA-seq data processing

Reads were downloaded from NCBI’s SRA archive (**Table 3**). They were then trimmed of poly-A, G, C, T tails and other lower-quality sequences using Trim Galore version 0.6.7 with its default options [18]. Single-cell UMAP was preprocessed using Seurat version 4.0.2 with default filters [19]. Runs were combined in Seurat following the “Introduction to scRNA-seq integration” vignette described on the package website.

**Table 3:** Details and BioProject accession number for each analyzed dataset.

BioProject No.	Virus	Tissue & Cell Type	Seq
PRJNA371753	HBV	Liver	Bulk
PRJNA644611	HIV	Monocytes (Innate Immune)	Single-cell
PRJNA639462	HIV	Frontal Cortex	Bulk
PRJNA521359 PRJNA448285	HIV	CD4^+^ T Cells (Adaptive Immune)	Bulk

This is a concise description of each analyzed dataset.

### Complexity analysis and dependencies

We performed runtime and memory analyses on downsampled HIV-infected T-cell dataset with 16 CPUs and 64 GB RAM. Runtime linearly depended on the number of reads, while memory remained constant at 30 GB, the size of the human genome (**S1 Fig**). A short list of Venus’s software dependencies includes STAR, Samtools, and Numpy, but a full list can be found on our GitHub page. For hardware dependencies, Venus needs to have a writing disk space of 100GB while around 30GB for RAM, ideally with at least 8 parallel threads for timely analysis.

## Results

### Venus accurately detected HBV-infection and fusion sites in patients with hepatocellular carcinoma

**Fig 3:**
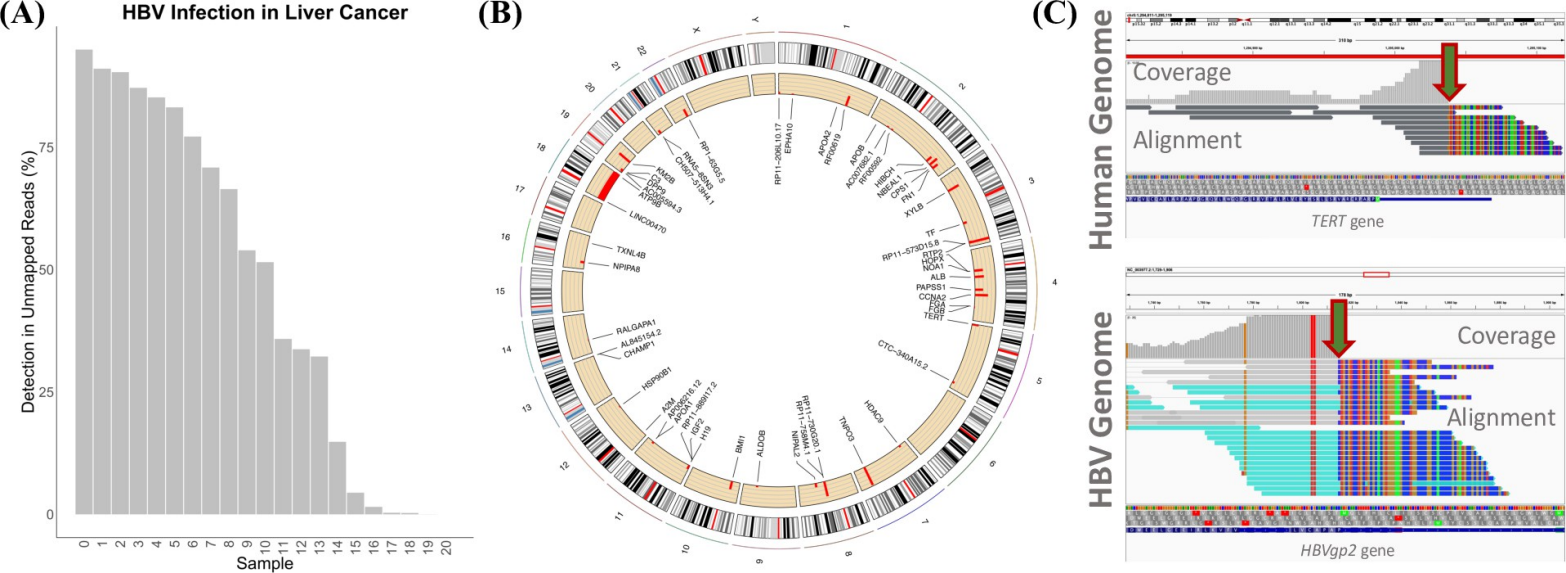
HBV viral detection and integration. **(A)** Percentage of unmappable human reads that mapped to HBV **(B)** Circos plot of detected fusion sites **(C)** Chimeric breakpoint between HBV *gp2* and human *TERT* visualized in IGV.

Due to the well-documented association of HBV-infection and liver cancer, we first applied Venus on HBV-infected liver cancers to detect viral load and integration sites. HBV has been heavily implicated in liver cancer due to its disruption of host DNA after viral integration events [20]. Utilizing the default parameters (see details in methods), Venus successfully detected HBV infection in 20 out of 21 patients undergoing surgery for hepatocellular carcinoma (**Fig 3A)**. The number of mappable reads ranged from 1 (sample 19) to 30,769 (sample 0), with an average of 46.4% reads that have been mapped. The fraction is out of all the reads that have been mapped to the mega-viral genome containing multiple viral species, which usually represent only 1 to 10% of reads unmapped to the human genome. We found that among all reads that had mapped to the mega-virus, HBV was frequently the top hit (**S1 Table**). These robust percentages and mapping results demonstrate Venus’s accuracy in detecting viral infection.

After confirming the detection of HBV in most samples, we ran the integration site discovery module to locate HBV fusion sites in the human genome. Interestingly, we detected 52 HBV fusion sites over 18 chromosomes (**Fig 3B**). We specifically examined a chimeric breakpoint–with 10 supporting transcripts–between HBV *gp2* and human *TERT*, a major oncogene and a documented integration site (**Fig 3C**) [21]. The red-green arrows point to sharp cuts where the alignment switched from human to HBV. The single colors indicate well-matched portions, while the multi-colors indicate reference-diverging portions. The sharp junction gave us high confidence that we had indeed detected a chimeric breakpoint. In fact, *Gp2*’s oncogene disruption has been widely cited as one of the many broken checkpoints leading to liver cancer [22]. In detecting integration sites, Venus provided a more detailed reason for this patient’s cancer diagnosis beyond the vague explanation of HBV infection.

### Venus precisely identified HIV-infected cells at a single-cell resolution in monocytes at various stages of maturity

**Fig 4:**
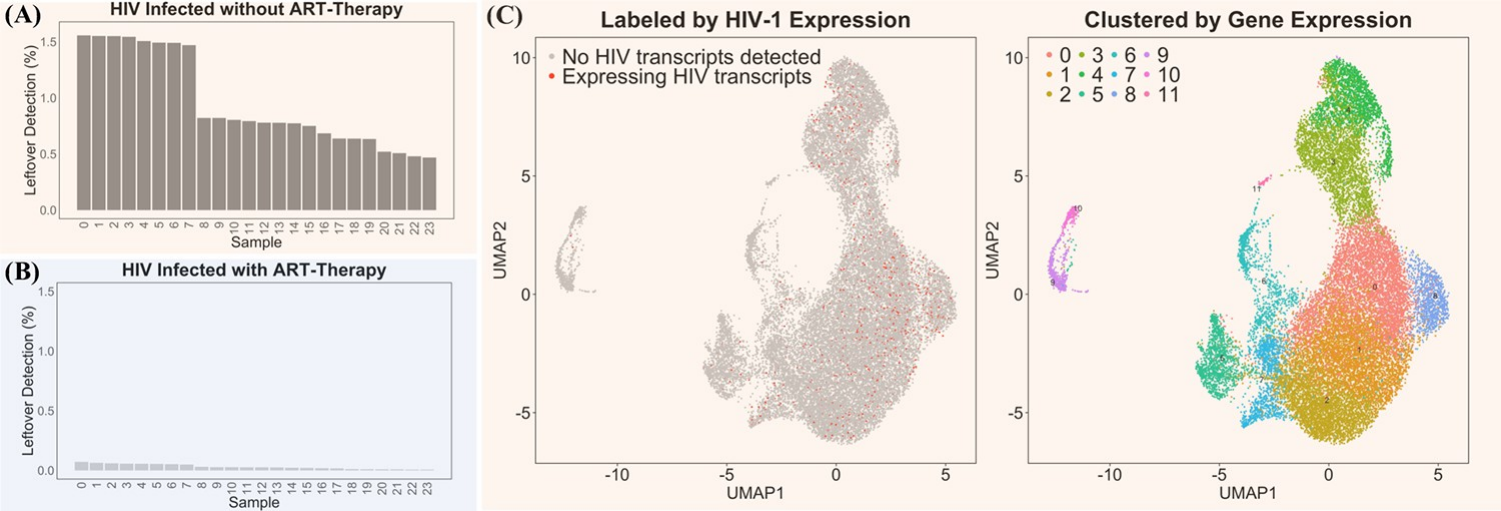
Venus’s single-cell analysis of HIV infection. **(A)** Percentage of unmappable human reads that mapped to HIV in HIV-infected (treatment one) **(B)** Percentage of unmappable human reads that mapped to HIV in HIV-infected, ART-treated (treatment two) **(C)** UMAP Left: labeled by HIV expression; Right: clustered by gene expression.

We further demonstrated Venus’s single-cell capability by analyzing a HIV-infected single-cell dataset, which had 8 uninfected samples as controls, 24 HIV-infected as treatment one, and another 24 HIV-infected but AntiRetroviral Therapy-treated (ART) as treatment two [23]. As expected, Venus found no viral load in all control samples, high viral load in treatment one (**Fig 4A**), and low viral load in treatment two (**Fig 4B**). Non-ART treated patients had a range of 531 to 2670 HIV transcripts, significantly higher than those from ART-treated patients with 7 to 198 HIV transcripts. Expectedly, ART treatment significantly suppressed viral load, exhibiting Venus’s accurate detection capability in a single-cell setting.

To visualize Venus’s single-cell capability, we labeled each infected cell with Venus-generated output (**S1 File**) to produce a UMAP plot in Seurat (**Fig 4C**) [24]. Out of the 25,211 cells that had passed Seurat’s default filters, 1056 cells harbored HIV transcripts. And after clustering, 12 different gene-expression groups of monocytes were found [9]. While there was no preference of infection toward any of the 12 different clusters, it exhibits Venus’s capability to provide a single-cell resolution picture of viral infection. We want to clarify to the readers that Venus is a computational pipeline that outputs viral-infected reads and integration sites with a minimal role in deciding single-cell processing parameters. However, our pipeline allows for two modes of sensitivity to let the users decide which mode best suit their analysis’s purpose (**S2 Fig**). Using random sampling, we also simulated the event of dropout common to single-cell sequencing in a bulk dataset with high viral load (HBV infection in liver cancer) and found that dropout linearly affected the viral detection rate, with a varying number of reads due to the sampling nature of sequencing experiments (**S3 Fig**). Finally, our pipeline has included statistical quantification of viral transcripts for statistical rigor (**S1 Eqn**).

### Venus detected HIV transcripts in the novel target frontal cortex beyond the blood-brain barrier

Historically, the frontal cortex was considered to be unreachable by viruses due to the blood-brain barrier [25]. However, recent literature have suggested that HIV could infect the human brain and result in a latent reservoir for the persistent HIV/AIDs disease [26]. To test this theory, we downloaded and analyzed a dataset originating from HIV-infected patients who had neurological deficiencies. Some were deemed cognitively normal (CN), while others were further differentiated based on day-to-day functional status: asymptomatic (ANI), minor disorder (MND), or dementia (HAD) [27].

**Fig 5:**
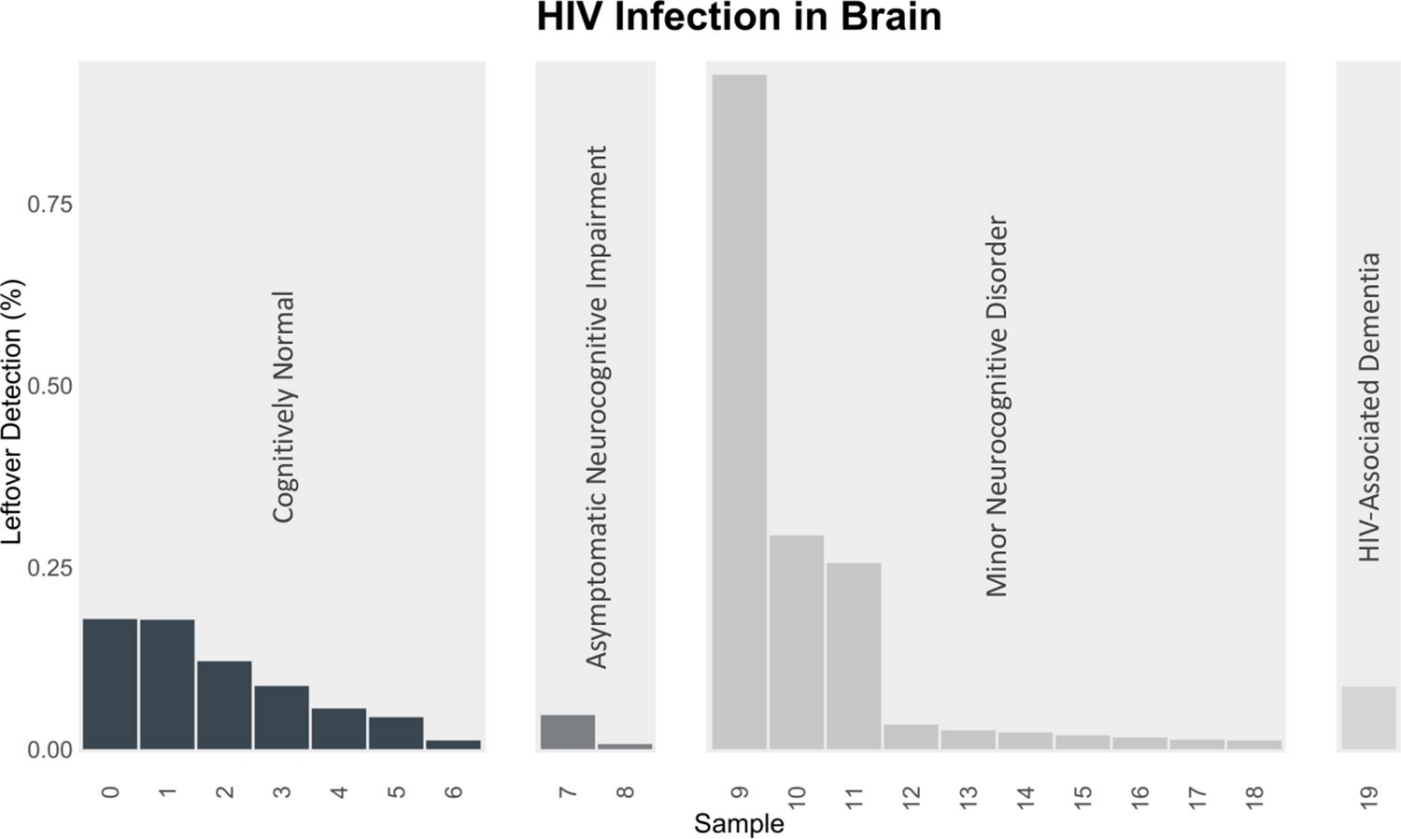
HIV detection behind the blood-brain barrier. Percentage of unmappable human reads that mapped to HIV from those who were deemed cognitively normal (CN), asymptomatic (ANI), minor disorder (MND), or dementia (HAD).

Out of the 41 HIV-infected frontal cortices, Venus detected transcripts in 20 or half of them (**Fig 5**). CN had a mean of 9.4 transcripts, ANI 4.5 transcripts, MND 16.2 transcripts, and HAD had 37 transcripts. Notably, over 100 HIV transcripts were found in sample 9. We discovered a small positive correlation between the severity of neurocognitive impairment and the number of detected HIV transcripts (Pearson correlation = 0.126). The discovery of viral infection in the hard-to-reach and previously-thought viral-free frontal cortex demonstrates Venus’s capability to detect infection in novel targets.

### Venus discovered HIV integration sites with varying biological significance and confidence in T-cells

**Fig 6:**
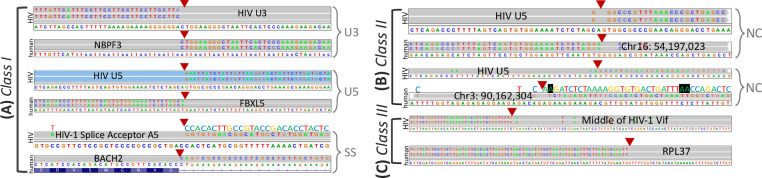
Venus’s classification of integration sites on HIV. Reference sequences of each species are at the bottom of each read. Due to converging HIV and human gene orientations, some sequences require reading their complements, written above in colorful letters. **(A)** Class I Integration Sites: human reading into HIV U3 sequence (U3), HIV U5 reading into human sequences (U5), or known splice sites from both species (SS) **(B)** Class II Integration Sites: U5 or U3 sequences that read into noncoding human regions (NC), differentiated by genomic coordinates **(C)** Class III Integration Sites: sites mapped to the middle of HIV genes.

Lines of literatures have highlighted the importance of virus integration sites due to their strong linkage to viral persistence, especially in the incessant HIV/AIDs epidemic [28]. Despite this, integration sites are often falsely concluded due to library preparation and sequencing artifacts [29]. To address these challenges, Venus classified HIV fusion transcripts into three categories based on biological relevance (see details in methods): Class I) fusion sites with human sequence reading into HIV’s U3 sequence, HIV’s U5 reading into human sequence, or splice donor-acceptor pairs (**Fig 6A**); Class II) fusion sites with the aforementioned sequences but reading into noncoding human regions (**Fig 6B**); Class III) fusion sites mapped to the middle of HIV genes (**Fig 6C**).

In the HIV-infected T-cells dataset, Venus found 17 Class I (**S4–S6 Figs**), 2 Class II, and 6116 Class III integration sites. We were confident that the first two classes of fusion sites were integration sites because of three telltale signs in **Fig 6**: 1) Unmatched sequences overlay perfectly onto the opposite specie’s reference; 2) Reads switch sharply in the middle between species, labeled by the red triangle breakpoints; 3) Nucleotides match the canonical U3 and U5 sequences used in HIV’s integration events [16,30,31]. Indeed, all three signs together showed that biologically-accurate integration sites were detected. Integration sites are inherently very difficult to detect, requiring a sequencing depth of 10X coverage [5]. While it may be interesting to compare across datasets, of the three HIV datasets studied, namely brain, monocytes, and T cells, only T cells were sequenced deeply enough to detect such integration sites.

While both Venus’s integration site classification algorithm and visualization capability were used to obtain high-confidence integration sites, they were also used to discard biologically irrelevant fusion sites. In contrast to Class I and IIs, Class IIIs likely signified partial integrations and sequencing artifacts due to their HIV gene disruptions. With the guide integrSeq parameter and subsequent visualization in IGV, Venus reduced the large amount of noise inherent to viral integration site discovery. We have provided a visualization capability in Venus because we understood viral integration events may vary from virus to virus, thus wishing to rest the final decision to each user [12]. In conclusion, not only could Venus detect chimeric fusion transcripts but also was it able to classify them into biologically meaningful integration sites.

### Availability and future directions

Venus is an open-source software package that can be freely downloaded at https://github.com/aicb-ZhangLabs/Venus. It leverages the recent single-cell sequencing revolution to provide a high-resolution picture of viral infection and integration sites. Venus is highly efficient with a linear increase in runtime and constant in memory consumption. It is worth mentioning that virus detection with RNA-seq data is still challenging for various reasons. For instance, if a virus’s target cell type is rare, the detection rate can be low due to difficulties in capturing such cells and the sparsity in single-cell sequencing. With the recent technology advances and data initiatives, we anticipate that the number of datasets will exponentially increase. Thus, multi-sample virus detection will improve the detection efficiency in rare cell types. Adding on, Venus mainly targets integration sites in the transcribed regions, leaving it challenging for non- transcribed region site detection. This can be resolved in the future as we plan to extend our method into DNA-based sequencing technologies as well. With the explosion of sequencing data across tissues and viruses, we hope our pipeline will become a valuable tool in facilitating future viral data analysis.

## Supporting information

S1 Fig Runtime and Memory Analysis of Venus’s 2 Modules. (TIFF)

S2 Fig Sensitivity option in Venus. (TIF)

S3 Fig Simulation of dropout event in HBV infection of liver cancer. (TIF)

S4 Fig Class I integration sites with HIV-1 U5 sequence “…TCTCTAGCA”. There were 12 found in total. Black highlights indicate minor mismatches with LTR, which could be due to variants or sequencing errors. Due to converging HIV and human gene orientations, some sequences require reading their complements, written above in colorful letters.
(TIFF)

S5 Fig Class I integration sites with HIV-1 U3 sequence “TGGAAGGGC…”. There was only one found.
(TIFF)

S6 Fig Class I Integration Sites with canonical donor-acceptor splicing pairs. These were manually selected from Venus’s visualization file.
(TIFF)

S1 Table Top 3 hits for the 21 HBV-infected patients when mapped to the mega-virus. These numbers represent runs (patients) and there were 21 runs (patients) in total in this study.
(DOCX)

S1 Eqn Statistical analysis equation for transcript quantification. (DOCX)

S1 File Infected cell barcodes of HIV-infected monocytes. (TSV)

S2 File Details on Testing and Test Data. (DOCX)

S3 File Software code for Venus as a 7z archive. The same information can be found as well by following the tutorial posted in https://github.com/aicb-ZhangLabs/Venus.git.
(7Z) Click here for additional data file.

S4 File Parameters and documentation for Venus. The same information can be found as well by following the tutorial posted in https://github.com/aicb-ZhangLabs/Venus.git.
(PDF)
