# Supplementary figures and images for: Venus: An efficient virus infection detection and fusion site discovery method using single-cell and bulk RNA-seq data
Source: PLoS Comput Biol. 2022 Oct 27;18(10):e1010636. doi: 10.1371/journal.pcbi.1010636 (PMC9642901; doi:10.1371/journal.pcbi.1010636)

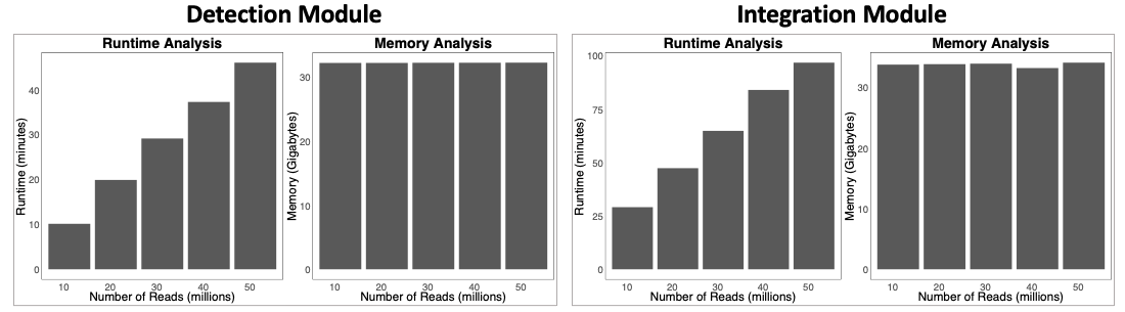

Supplement: S1 Fig — (TIFF) [file pcbi.1010636.s001.tiff]

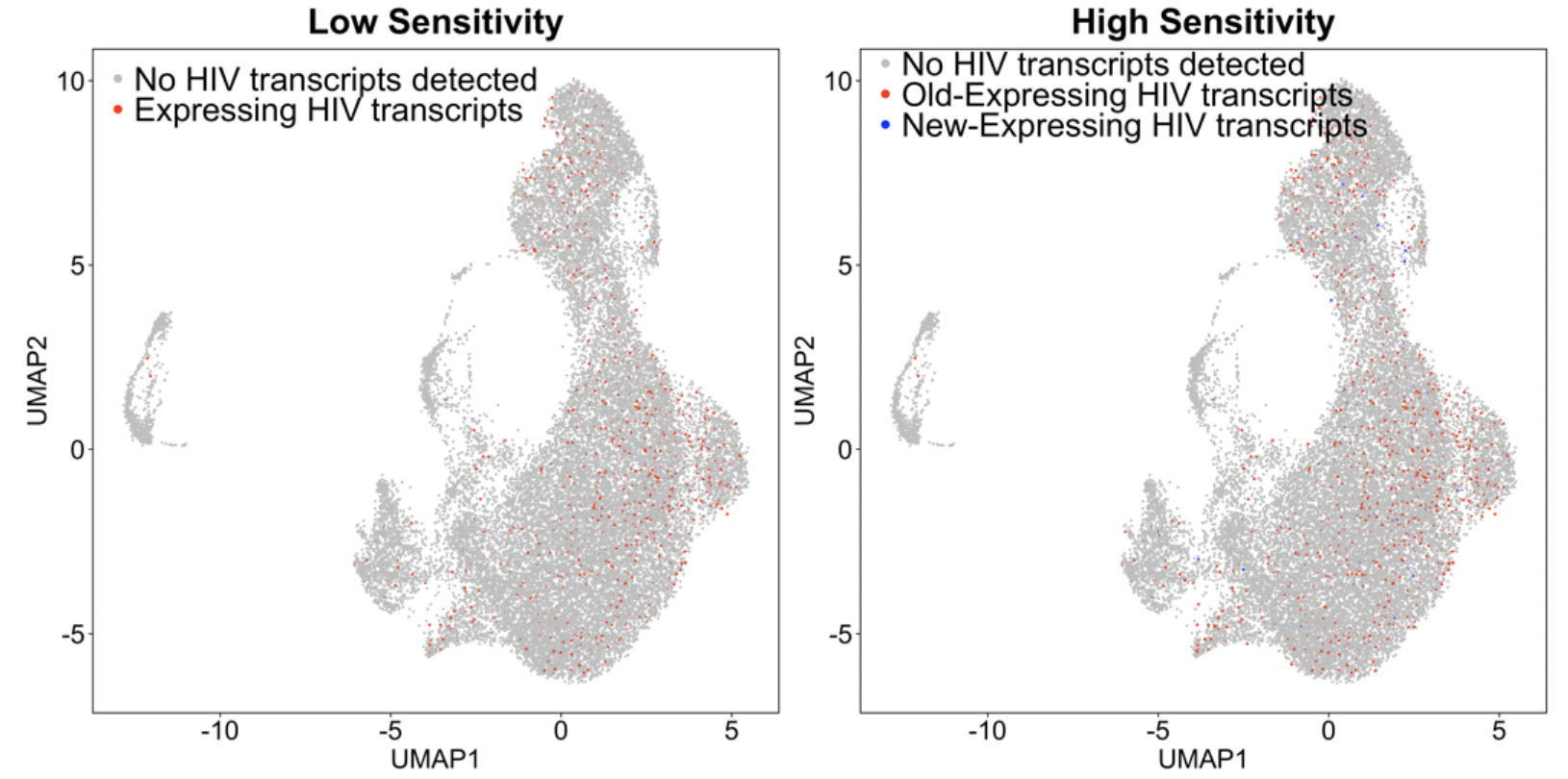

Supplement: S2 Fig — (TIF) [file pcbi.1010636.s002.tif]

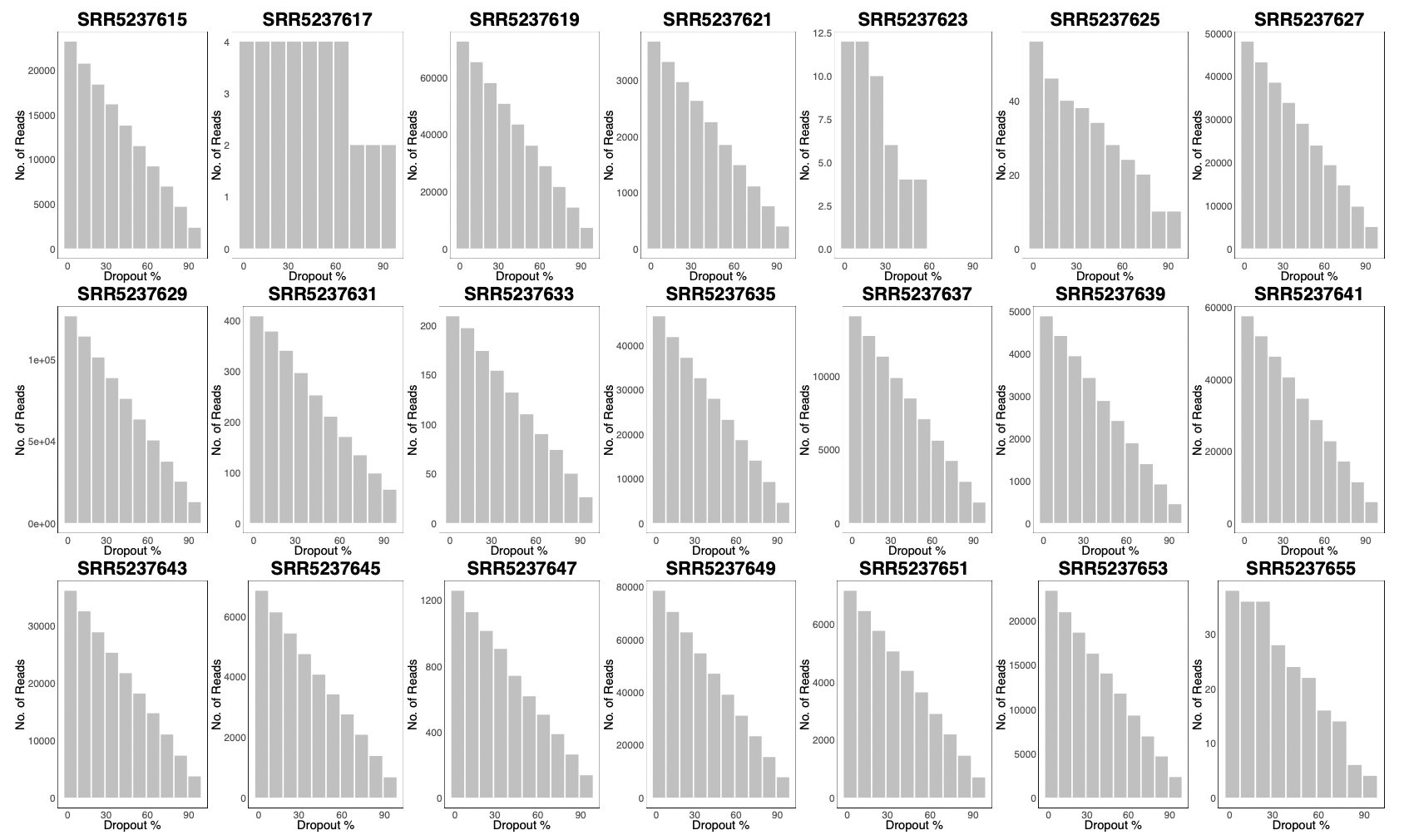

Supplement: S3 Fig — (TIF) [file pcbi.1010636.s003.tif]

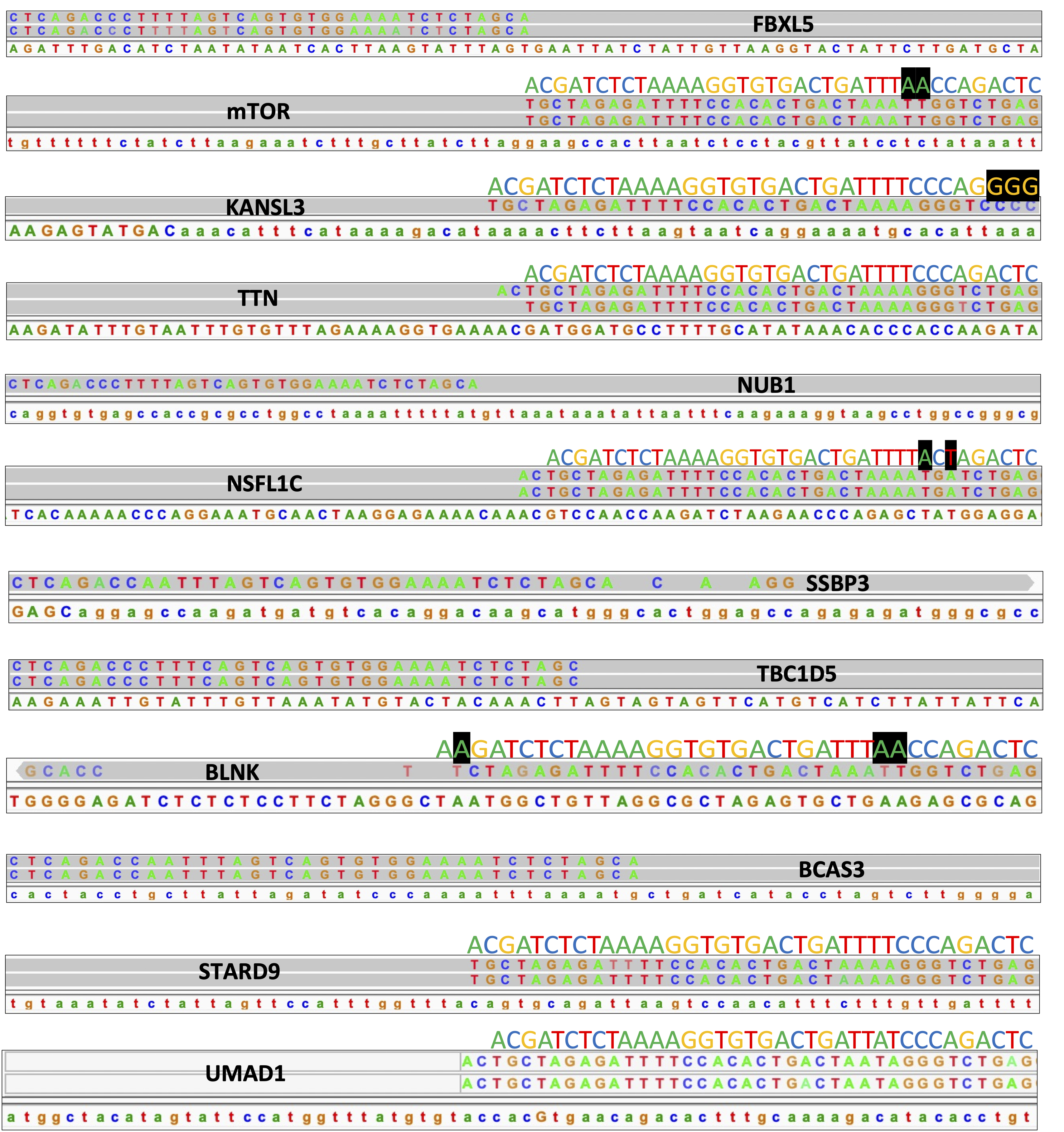

Supplement: S4 Fig — There were 12 found in total. Black highlights indicate minor mismatches with LTR, which could be due to variants or sequencing errors. Due to converging HIV and human gene orientations, some sequences require reading their complements, written above in colorful letters. (TIFF) [file pcbi.1010636.s004.tiff]

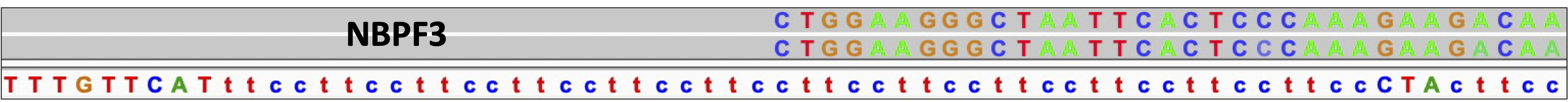

Supplement: S5 Fig — There was only one found. (TIFF) [file pcbi.1010636.s005.tiff]

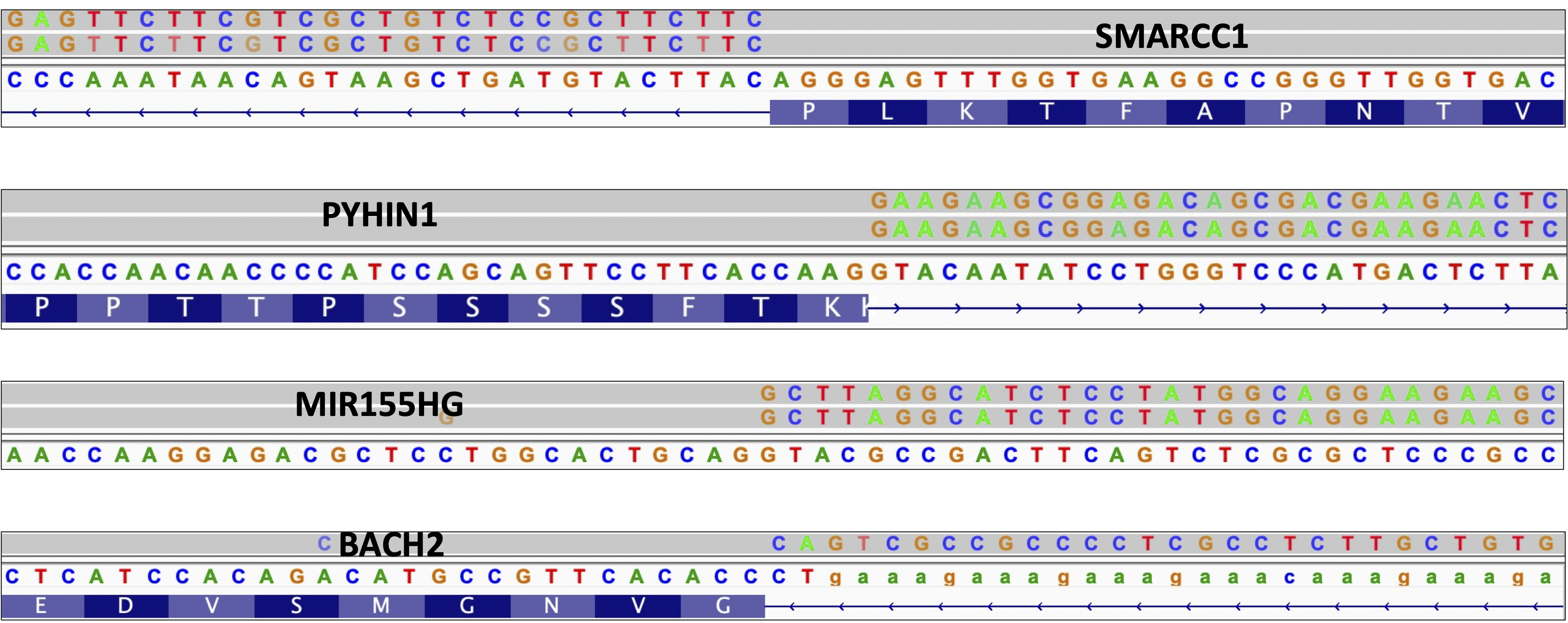

Supplement: S6 Fig — These were manually selected from Venus’s visualization file. (TIFF) [file pcbi.1010636.s006.tiff]
