# Supplementary material for: Venus: An efficient virus infection detection and fusion site discovery method using single-cell and bulk RNA-seq data
Source: PLoS Comput Biol. 2022 Oct 27;18(10):e1010636. doi: 10.1371/journal.pcbi.1010636 (PMC9642901; doi:10.1371/journal.pcbi.1010636)
Supplement: S1 Table — These numbers represent runs (patients) and there were 21 runs (patients) in total in this study. (DOCX) [file pcbi.1010636.s007.docx]

|  | 1^st^ Hit | 2^nd^ Hit | 3^rd^ Hit |
| --- | --- | --- | --- |
| Hepatitis B virus | 11 | 4 | 3 |
| Enterobacteria phage phiX174 sensu lato^a^ | 10 | 11 | 0 |
| Human endogenous retrovirus K113 complete genome | 0 | 6 | 15 |
| BeAn 58058 virus | 0 | 0 | 3 |

^a^While Venus detected abundant “enterobacteria phage phiX174 sensu lato” transcripts, even more than those of Hepatitis B virus, the transcripts were from a bacteriophage, a virus that infects bacteria and cannot infect human cells. So they were not included in our consideration.
