# Supplementary material for: Venus: An efficient virus infection detection and fusion site discovery method using single-cell and bulk RNA-seq data
Source: PLoS Comput Biol. 2022 Oct 27;18(10):e1010636. doi: 10.1371/journal.pcbi.1010636 (PMC9642901; doi:10.1371/journal.pcbi.1010636)
Supplement: S1 Eqn — (DOCX) [file pcbi.1010636.s008.docx]

$$\tau_{i}=\frac{\frac{\eta_{i}}{\tilde{l_{i}}}}{\sum_{j=1}^{M} \frac{\eta_{j}}{\tilde{l_{j}}}} \mathbf{S1 Eqn}$$

Specifically, $\tau_{i}$ is the expected number of transcripts from transcript $i$. $\eta_{i}$ is the total fraction of all nucleotides in the sample that originate from a copy transcript $i$. $M$ indicates the number of total unique transcripts. All values are normalized to transcript length $\tilde{l}$.
