# Supplementary material for: Venus: An efficient virus infection detection and fusion site discovery method using single-cell and bulk RNA-seq data
Source: PLoS Comput Biol. 2022 Oct 27;18(10):e1010636. doi: 10.1371/journal.pcbi.1010636 (PMC9642901; doi:10.1371/journal.pcbi.1010636)
Supplement: S2 File — (DOCX) [file pcbi.1010636.s010.docx]

**Details on Testing and Testing Data**

Details of how to install and run the software are all described in the README.md file in <https://github.com/aicb-ZhangLabs/Venus>. Documentation and expected output files are posted along the tutorial steps in the code repository.

The bulk and single-cell test data came from HIV-infected T-cells and monocytes, respectively, accession number PRJNA448285 and PRJNA644611. They were from the same data analyzed in the results sections (**Fig. 4, 6**), except that the test data were down-sampled so that the file sizes were less than 100 MB, as required by GitHub. Reference sequences for both human and viruses were downloaded from NCBI’s RefSeq database.
