# Supplementary material for: Venus: An efficient virus infection detection and fusion site discovery method using single-cell and bulk RNA-seq data
Source: PLoS Comput Biol. 2022 Oct 27;18(10):e1010636. doi: 10.1371/journal.pcbi.1010636 (PMC9642901; doi:10.1371/journal.pcbi.1010636)
Supplement: S4 File — The same information can be found as well by following the tutorial posted in https://github.com/aicb-ZhangLabs/Venus.git. (PDF) [file pcbi.1010636.s012.pdf]

Creating Index

module-index.py

Description

This step creates the necessary mapping indices directories in Venus, namely for the virus, human, and hybrid genomes.

Parameters

- humanFASTA: the FASTA reference file for human genome
- humanGTF: the GTF annotation file for human genome
- virusFASTA: the FASTA reference file for virus genome
- virusGTF: the GTF annotation file for virus genome (Optional)
- module: which module, detection or integration, we are generating the indices for
- thread: parallelization feature (Optional)
- hGenome: output index directory path for either the human or hybrid genome
- virusGenome: output index directory path for virus genome

Output

- out: path for the extra output files. Default is current working directory. (Optional)
- hGenome: index directory path for either the built human or hybrid genome
- virusGenome: index directory path for the built virus genome

Detection Module

module-detection.py

Description

This module detects viral load in the supplied reads and will output a list of infecting viral species (with infected cell barcodes if single-cell).

Parameters

- read: sequencing reads in fastq or fastq.gz formats. Please separate paired-end reads with white spaces.
- virusThreshold: minimum number of unique viral transcripts to count viral species infection (Optional)
- virusChrRef: a table of viral reference sequences' assembly name to species name
- virusGenome: index directory path for the built virus genome
- humanGenome: index directory path for either the built human genome
- readFilesCommand: commands necessary to uncompress gzipped reads (Optional)
- thread: parallelization feature (Optional)
- singleCellBarcode: for single-cell reads only, two integers specifying the start position and length of the cell barcode (Optional)
- singleUniqueMolIdent: for single-cell reads only, two integers specifying the start position and length of the UMI (Optional)
- singleWhitelist: for single-cell reads only, barcode whitelist (Optional)

Output

- out: path for the output files. Default is current working directory. (Optional)
    - detection_output.tsv: a list of infecting viral species (with infected cell barcodes if single-cell)

Integration Module

module-integration.py

Description

This module detects viral integration sites with a hybrid index. After classifying fusion transcripts into 3 classes of integration gene sites, it will output the first two most likely classes of genes in addition to a visualization file.

(For single-cell sequencing, please simply treat the cDNA read as a single-end sequencing experiment.)

Parameters

- read: sequencing reads in fastq or fastq.gz formats. Please separate paired-end reads with white spaces.
- virusGenome: index directory path for the built virus genome
- hybridGenome: index directory path for either the built hybrid genome
- guideFASTA: file containing sequences used to classify integration sites
- readFilesCommand: commands necessary to uncompress gzipped reads (Optional)
- virusChr: specifies the assembly name for a given viral species
- thread: parallelization feature (Optional)
- geneBed: file used to convert genomic coordinates to gene names

Output

- out: path for the output files. Default is current working directory. (Optional)
    - class1_integration.tsv: class 1 viral integration sites
    - class2_integration.tsv: class 2 viral integration sites
    - visual.bam, visual.bam.bai: files to visualize fusion transcripts in IGV
